# A Gadolinium(III) Complex Based on Pyridoxine Molecule with Single-Ion Magnet and Magnetic Resonance Imaging Properties

**DOI:** 10.3390/ijms25042112

**Published:** 2024-02-09

**Authors:** Marta Orts-Arroyo, Amadeo Ten-Esteve, Sonia Ginés-Cárdenas, Leonor Cerdá-Alberich, Luis Martí-Bonmatí, José Martínez-Lillo

**Affiliations:** 1 Departament de Química Inorgànica, Instituto de Ciencia Molecular (ICMol), Universitat de València, c/Catedrático José Beltrán 2, Paterna, 46980 Valencia, Spain; marta.orts-arroyo@uv.es; 2 Radiology Department and Biomedical Imaging Research Group (GIBI230), La Fe University and Polytechnic Hospital and La Fe Health Research Institute, 46026 Valencia, Spain; amadeo_ten@iislafe.es (A.T.-E.); soniagibi230@gmail.com (S.G.-C.); leonor_cerda@iislafe.es (L.C.-A.); luis_marti@iislafe.es (L.M.-B.)

**Keywords:** gadolinium, vitamin B_6_, single-ion magnet, magnetic resonance, contrast agent, relaxivity

## Abstract

Pyridoxine (pyr) is a versatile molecule that forms part of the family of B vitamins. It is used to treat and prevent vitamin B_6_ deficiency and certain types of metabolic disorders. Moreover, the pyridoxine molecule has been investigated as a suitable ligand toward metal ions. Nevertheless, the study of the magnetic properties of metal complexes containing lanthanide(III) ions and this biomolecule is unexplored. We have synthesized and characterized a novel pyridoxine-based Gd^III^ complex of formula [Gd^III^(pyr)_2_(H_2_O)_4_]Cl_3_ · 2 H_2_O (**1**) [pyr = pyridoxine]. **1** crystallizes in the triclinic system and space group *P*ī. In its crystal packing, cationic [Gd(pyr)_2_(H_2_O)_4_]^3+^ entities are connected through H-bonding interactions involving non-coordinating water molecules and chloride anions. In addition, Hirshfeld surfaces of **1** were calculated to further investigate their intermolecular interactions in the crystal lattice. Our investigation of the magnetic properties of **1**, through ac magnetic susceptibility measurements, reveals the occurrence of a slow relaxation in magnetization in this mononuclear Gd^III^ complex, indicating an unusual single-ion magnet (SIM) behavior for this pseudo-isotropic metal ion at very low temperatures. We also studied the relaxometric properties of **1**, as a potential contrast agent for high-field magnetic resonance imaging (MRI), from solutions of **1** prepared in physiological serum (0.0–3.2 mM range) and measured at 3 T on a clinical MRI scanner. The values of relaxivity obtained for **1** are larger than those of some commercial MRI contrast agents based on mononuclear Gd^III^ systems.

## 1. Introduction

Pyridoxine is one of the most common active forms or vitamers of vitamin B_6_, which is an enzymatic co-factor involved in more than one hundred metabolic reactions, including carbohydrate, amino acid, and lipid metabolism in humans [1]. This molecule is necessary for normal brain function, given that it actively aids in producing neurotransmitters such as dopamine, serotonin, norepinephrine, and gamma-aminobutyric acid [2,3,4,5]. Vitamin B_6_ deficiency is associated with depression, convulsive seizures, mild microcytic hypochromic anemia, and calcium oxalate nephropathy [6]. 

**Scheme 1:**
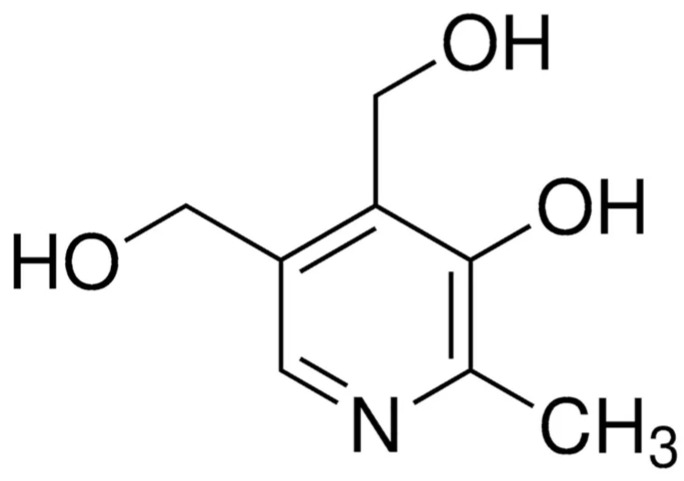
Molecular structure of pyridoxine (pyr).

Over more than four decades, pyridoxine has also been investigated as a suitable ligand toward the preparation of metal complexes. Its pyridinic nitrogen atom and alcohols groups have been used to coordinate several metal ions (Scheme 1), such as Cd^II^ [7], Fe^III^ [8], Co^III^ [9], Cu^II^ [9,10,11], Sn^IV^ [12,13,14], and U^VI^ [15,16,17,18,19,20]. In most cases, pyridoxine molecule chelates or bridges through its alcohols groups, in the form of alkoxide, phenolate, or just as alcohol functional groups, and only in a few systems the pyridinic nitrogen atom is involved in metal coordination [20,21,22,23]. From all this family of pyridoxine-based complexes, only a couple of systems have been investigated regarding their magnetic properties [18]. Concerning the Gd^III^ ion, there only exists one Gd^III^ complex based on pyridoxine which has been reported in the literature [24]. This compound, of formula [Gd^III^(pyr)_2_(NO_3_)_2_(H_2_O)](NO_3_), binds circulating tumor DNA (ctDNA) with a moderate affinity [24]. Nevertheless, no magneto-structural study on a pyridoxine-based Gd^III^ complex has been reported to date. 

When we compare the magnetic properties of Gd^III^ complexes with those of other lanthanide(III) complexes, we note that Gd^III^ complexes have been scarcely studied. The Gd^III^ ion has been investigated many times as a magnetically isotropic ion, given that it exhibits a half-filled 4f^7^ configuration with a lack in orbital contribution, that is, it has S = 7/2 and L = 0, respectively [25,26]. Thus, there exists a very low quantity of reported homometallic Gd^III^ species displaying slow relaxation of magnetization and single-molecule/ion magnet (SMM/SIM) [26,27,28].

On the other hand, Gd^III^ metal ion is also employed as a contrast agent in magnetic resonance imaging (MRI), to improve lesion detection and characterization, finally increasing the efficacy of diagnostic MR scans, given that Gd^III^ ion promotes changes in the relaxivity of protons from the associated coordination water molecules and generates a signal with clearer physical distinction among the contrast agent and the surrounding tissues. Thus, Gd^III^-based contrast agents have revolutionized modern technological advances in radiological diagnostics [29]. Nevertheless, novel Gd^III^-based contrast agents are being investigated to improve the response of the current 3 T scanner devices [29,30]. 

It is well known that the human body uses vitamin B_6_ in the metabolism. Therefore, it would be very interesting to obtain a complex based on Gd^III^ and vitamin B_6_, which could act as a process-specific contrast agent. 

Herein, we report the synthesis, the crystal structure and the magnetic and relaxometric properties of a novel pyridoxine-based Gd^III^ compound of formula [Gd^III^(pyr)_2_(H_2_O)_4_]Cl_3_·2H_2_O (**1**) [pyr = pyridoxine]. To our knowledge, **1** constitutes the first example of a gadolinium-based single-ion magnet (SIM), whose study on its magnetic resonance imaging properties in a 3 T scanner has been reported. 

## 2. Results and Discussion

### 2.1. Description of the Crystal Structure

**Figure 1:**
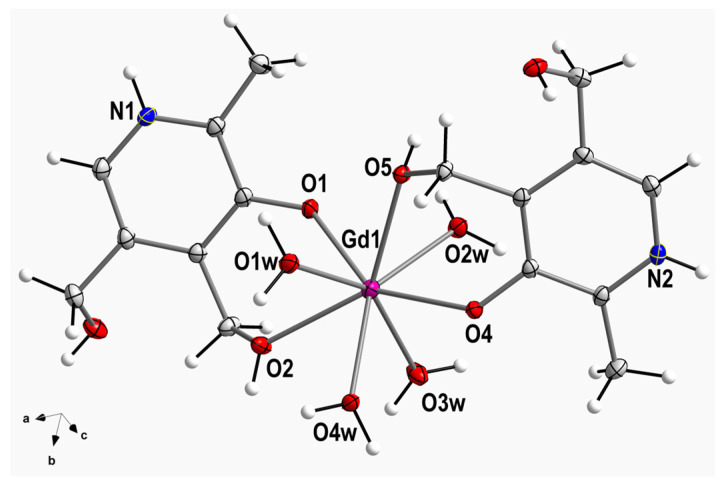
Molecular structure of the [Gd(pyr)_2_(H_2_O)_4_]^3+^ species in **1**. A selected atom numbering scheme along with thermal ellipsoids (50%) are also shown. Non-coordinated water molecules and chloride anions have been removed.

Compound **1** crystallizes in the triclinic system and space group *P*ī (Table 1). The crystal structure is made up of cationic mononuclear [Gd^III^(pyr)_2_(H_2_O)_4_]^3+^ complexes, chloride anions, and crystallization water molecules. Indeed, the asymmetric unit of **1** contains a [Gd^III^(pyr)_2_(H_2_O)_4_]^3+^ complex, three chloride anions, and two water molecules. A perspective drawing showing the cationic Gd^III^ complex in **1** is given in Figure 1. In compound **1**, the Gd^III^ metal ion is eight-coordinate and bonded to eight oxygen atoms, two oxygen atoms from alcohol groups (O2 and O5), two oxygen atoms from phenolate groups of two pyridoxine ligands (O1 and O4) and four oxygen atoms of four water molecules (O1w–O4w) (Figure 1). Considering the Gd-O bond lengths involving alcohol groups, they show an average value of 2.360(1) Å, which is somewhat shorter than that of the Gd-O bond lengths of water molecules [2.424(1) Å] (Table S1). 

The O-Gd-O bond angles present values which cover a range from 69.13(4) to 148.95(4)°. In **1**, the two pyridine rings are planar and form an intramolecular angle of ca. 17.7(1)°. The CߝC, CߝN, and CߝO bond lengths agree with those found in the literature for the pyridoxine molecule coordinated to different metal ions (Table S1) [8,9,10,11,12,13,14,15,16,17,18,19]. All these crystallographic data agree with those reported for other similar compounds [27,30,31].

**Figure 2:**
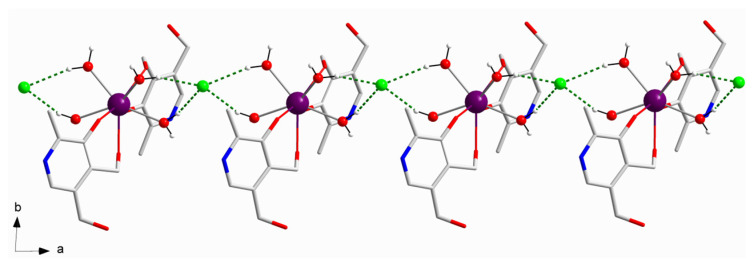
View of the one-dimensional motif obtained by H-bonding interactions between chloride anions and water molecules of [Gd(pyr)_2_(H_2_O)_4_]^3+^ complexes in **1**. Selected H atoms and non-coordinated water molecules are omitted for clarity. Color code: purple, Gd; green, Cl; red, O; blue, N; grey, C; white, H.

**Figure 3:**
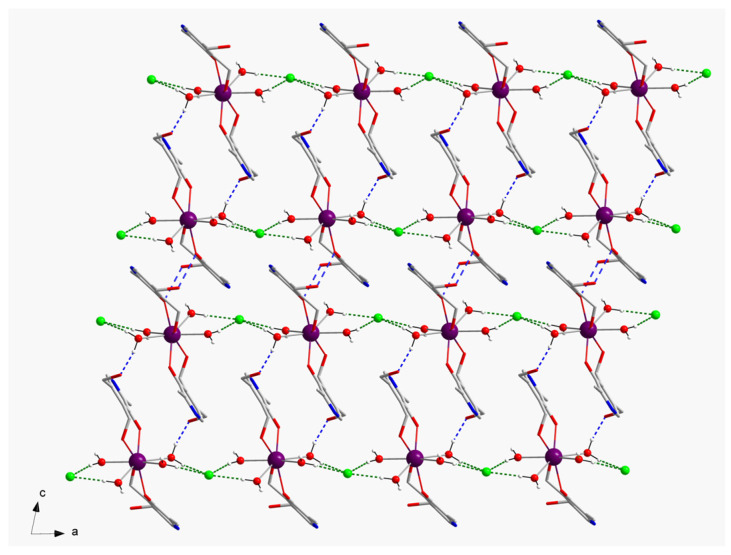
View of a fragment of the supramolecular 2D network generated by H-bonded O···O (dashed blue lines) and Cl···O (dashed green lines) interactions, which grow along the crystallographic ac plane in **1**. Selected H atoms and non-coordinated water molecules are omitted for clarity.

In the crystal of **1**, the cationic [Gd(pyr)_2_(H_2_O)_4_]^3+^ entities are connected by means of H-bonding interactions generated by coordinated-water molecules and chloride anions, thus generating a 1D motif that grows along the crystallographic a axis [O1w···Cl2a = 3.133(1) Å and O4w···Cl2a = 3.147(1) Å; (a) = x + 1, y, z] (Figure 2). Additional H-bonding interactions, which are formed through coordinated-water molecules and alcohol groups of pyridoxine ligands of adjacent Gd^III^ complexes, result in a layered structure growing in the ac plane [O3···O2wb = 2.730(2) Å; (b) = −x + 1, −y + 2, −z] (Figure 3). The shortest intermolecular Gd···Gd separation in **1** is approximately 8.640(1) Å [Gd1···Gd1c; (c) = −x + 1, −y + 2, −z + 1]. Finally, the -NH groups of pyridoxine molecules and chloride anions are H-bonded in the third dimension of the crystal structure of **1** [N1···Cl3d = 3.218(2) Å; (d) = x + 1, y, z − 1] (Table 2). 

We further analyzed the coordination environment and geometry of the Gd^III^ ion in **1**. For doing that, the SHAPE program was employed. This program allows us the calculation of different polyhedra and molecular geometries for metal complexes, with the 0.000 value being the perfect match for the ideal or regular polyhedron [32]. The resulting metal symmetry can be compared with previously reported complexes and their magnetic properties [33]. In **1**, the single Gd^III^ ion displays a coordination number (CN) equal to 8. The lowest SHAPE value obtained for **1** was 0.948, which was assigned to the triangular dodecahedron geometry (TDD), the second lowest value being 1.441, which was associated with a square antiprism geometry (SAPR), see Table 3. Thus, these calculations allow us to assign the D_2d_ symmetry to the Gd^III^ metal ion in compound **1** (Table 3).

### 2.2. Analysis of the Hirshfeld Surfaces

**Figure 4:**
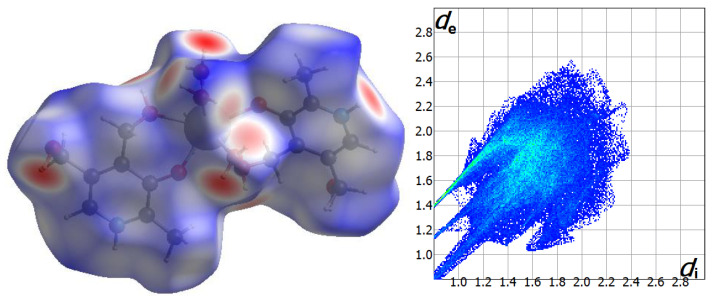
Hirshfeld surface mapped through d_norm_ function for the mononuclear Gd^III^ complex obtained with pyridoxine molecule in **1** (**left**); Full fingerprint plot for compound **1** (**right**).

Intermolecular interactions involving the cationic [Gd(pyr)_2_(H_2_O)_4_]^3+^ complex of **1** were further investigated by means of CrystalExplorer program [34]. The qualitative and quantitative investigation of the main intermolecular contacts was performed by mapping the distances of the 3D surface generated considering the nearest atom outside (d_e_) and inside (d_i_) distances of **1** and a normalized contact distance (d_norm_), which overcomes some limitations because of different atom sizes [35,36,37]. A redߝwhiteߝblue set of colors is used for assigning shorter contacts (red), contacts around the van der Waals separation (white), and those longer contacts (blue) [34]. Moreover, a 2D plot of the involved intermolecular interactions is generated as a fingerprint [34,35,36,37]. The Hirshfeld surface and the fingerprint plot for the cationic unit of compound **1** are given in Figure 4 and Figure S1.

The intermolecular H···Cl contacts involving mainly the coordinated water molecules, and also the -NH group of the pyridine ring of pyridoxine molecules, and chloride anions are the main interactions which are reflected on the fingerprint plot with ca. 26% (Figure 4). An important part of these interactions is responsible for generating the 1D motifs and also the final 3D structure formed by the [Gd(pyr)_2_(H_2_O)_4_]^3+^ cations and Cl^−^ anions in **1**. Moreover, intermolecular H···O contacts involving non-coordinated water molecules, coordinated water molecules, and different protonated and deprotonated alcohol groups of pyridoxine ligands connect adjacent Gd^III^ complexes through H-bonding interactions, generating a layered structure. These H···O contacts are approximately 18% of the complete fingerprint plot (Figure 4). 

### 2.3. Magnetic Properties Study

**Figure 5:**
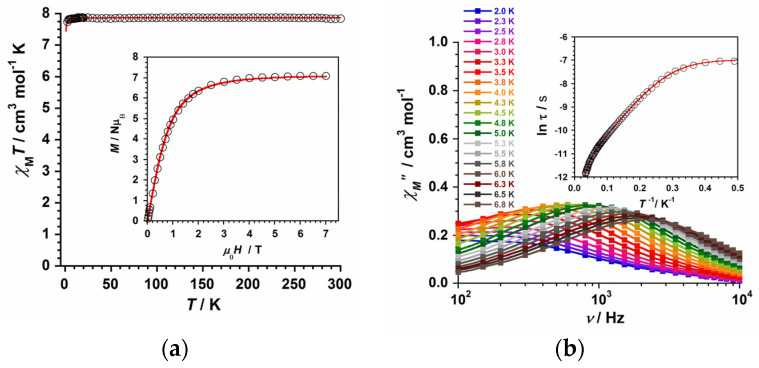
(**a**) χ_M_*T* product versus *T* plot for compound **1**, the solid red line representing the theoretical fit of the experimental magnetic susceptibility data. The inset shows the field dependence of the molar magnetization at 2.0 K (the solid red line representing the theoretical fit through the Brillouin curve generated with *g* = 2.0 and S = 7/2); (**b**) Frequency dependence of the out-of-phase ac susceptibility signals under a dc field of 2500 G for **1**. The inset displays the ln(τ) vs. 1/*T* plot with the fit considering the contribution of two Orbach + quantum tunnelling of magnetization (QTM) mechanisms.

The study of the magnetic properties of compound **1** was carried out through direct current (dc) and alternating current (ac) magnetic susceptibility measurements. During the dc measurements, eicosene was used to immobilize the investigated sample. In Figure 5a, the experimental χ_M_*T* vs. *T* plot for **1** is shown, which was obtained through an external magnetic field of 0.5 T and a temperature range of 2–300 K. At *T* = 300 K, the χ_M_*T* value is equal to 7.84 cm^3^mol^−1^K, which is just about that expected for one Gd^III^ metal ion (4f^7^ ion with *g* = 2.0, S = 7/2 and L = 0) [25,26,27,28]. Below 300 K and upon cooling, the χ_M_*T* value keeps constant with decreasing temperature to ca. 6.0 K. Below that temperature, the χ_M_*T* value decreases slowly reaching a minimum value of approximately 7.69 cm^3^mol^−1^K at 2.0 K. These dc magnetic susceptibility results, which were obtained at very low temperatures, would be interpreted as an effect of intermolecular interactions and/or a very small zero-field splitting (ZFS) taking place in compound **1** [25,26,27,28].

We have also measured the field dependence of the molar magnetization (M) for **1** in the 0–7 T range (see inset in Figure 5a). Our experimental values followed a constant increase in M with the magnetic field at a fixed temperature (2.0 K). These values were well simulated through the Brillouin analytical expression, which was generated with *g* and S values of 2.0 and 7/2, respectively (Figure 5a). In this M vs. H curve, the largest experimental value for **1** was equal to 7.07 μ_B_ [25,26]. 

In order to treat the experimental susceptibility points of the χ_M_*T* vs. *T* curve of **1**, we employed the theoretical expression which is typically associate with an isotropic ion exhibiting a S value equal to 7/2. Moreover, we added an extra variable with a theta (θ) symbol, which can explain the intermolecular interactions observed in compound **1** [χ_M_ = (Nμ_B_^2^*g*^2^/3k_B_)S(S + 1)/(*T* − θ)] [30]. Finally, through least-squares fits, we obtained the following values for compound **1**: *g* = 2.005(1) and θ = −0.034(2) K (*R* = 5.3 × 10^−5^). 

In addition, alternating current (ac) magnetic susceptibility measurements were carried out on a sample of **1** in a 5.0 G ac field oscillating at different frequencies (10^2^–10^4^ Hz range) in the temperature range of 2.0–7.0 K. No slow relaxation of the magnetization was observed for the sample of **1** at H_dc_ = 0 G. However, out-of-phase ac signals (χ″_M_) were detected when an external dc magnetic field of 2500 G was applied on **1**, indicating a field-induced Single-Ion Magnet (SIM) behavior for this mononuclear Gd^III^ system [25]. This singular magnetic behavior of **1** was studied through its χ_M_″ vs. frequency (ν/Hz) plot, which is given in Figure 5b. From the measured data of the relaxation maxima in the χ_M_″ vs. frequency (ν/Hz) plot, the ln(τ) vs. 1/*T* curve is obtained, which is given in the inset of Figure 5b. The experimental data of the ln(τ) vs. 1/*T* curve draw up to three different sections, one straight line along the ca. 0.01–0.08 K^−1^ range, followed by another one in the ca. 0.10–0.30 K^−1^ range of the high-temperature region, and another straight line along the ca. 0.40–0.50 K^−1^ range of the low-temperature region. This singular ln(τ) vs. 1/*T* curve was fully fitted only when we considered two Orbachs plus a quantum tunnelling (QTM) mechanisms for the relaxation of magnetization in **1** [*τ*_o_^−1^_(1)_exp(−U_eff(1)_/k_B_*T*) + *τ*_o_^−1^_(2)_exp(−U_eff(2)_/k_B_*T*) + *τ*^−1^_QTM_]. The least-squares fit of these data led to the set of parameters: U_eff_ (1) = 63.9(1) cm^−1^, *τ*_o_ (1) = 7.9(1) × 10^−7^ s, U_eff_ (2) = 12.0(1) cm^−1^, *τ*_o_ (2) = 6.9(2) × 10^−6^ s and *τ*^−1^_QTM_ = 1069(5) s^−1^ for **1**. 

The relaxation dynamics that **1** exhibits as single-ion magnet (SIM) is somewhat different to those of other previously reported Gd^III^ systems, given that no Raman mechanism was extracted from the experimental ac data of **1**. Nevertheless, the reported effective energy barrier (U_eff_) values obtained for **1** should be carefully considered as they might not correspond a priori to any excited states of the Gd^III^ ion and, hence, they would not be real U_eff_ values [26,28,33]. In any case, compound **1** is one of the few SIMs based on Gd^III^ ion reported up to date, hence, further detailed experimental and theoretical investigations carried out on this quasi-isotropic 4f metal ion will be necessary to correctly understand the relaxation dynamics of the uncommon Gd^III^-based SIMs. 

### 2.4. MR Imaging Phantom Studies

We have also studied the relaxometric properties of **1**, as a potential contrast agent for high-field MRI [38,39,40,41,42,43,44,45]. A series of 13 samples of **1** (the concentrations ranging from 0.0 to 3.2 mM) were prepared in physiological serum and were measured on a clinical MR scanner (Achieva 3T TX, Philips Healthcare, Best, The Netherlands). These measurements were performed by placing the 13 samples (with range of pH values: 7.0–7.4) in a volumetric head eight channels SENSE coil and were monitored over a period of three weeks [30].

**Figure 6:**
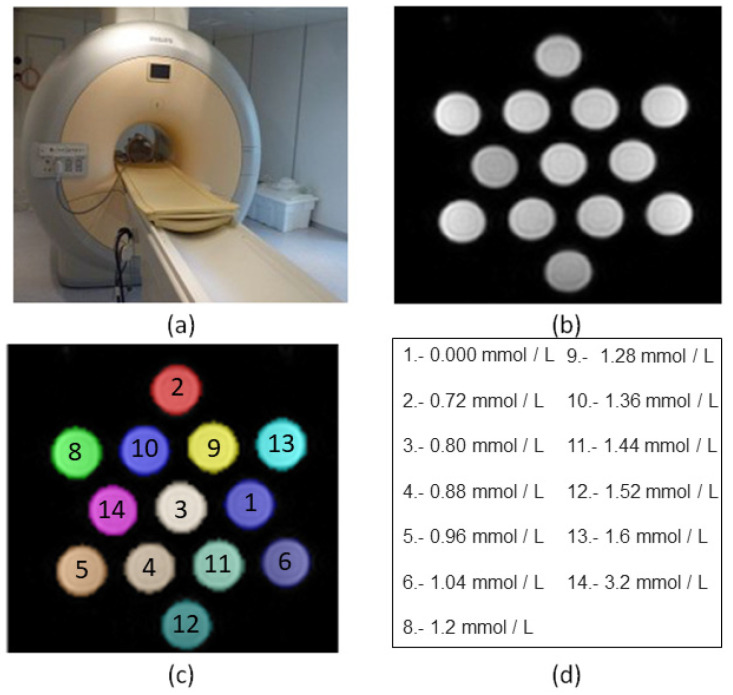
(**a**) MR imaging scanner (Philips Achieva 3T); (**b**) MR images of the tube phantoms containing concentrations of **1** covering the range of 0.0–3.2 mM in physiological serum, the image corresponds to the first echo of a cross section of the multi echo gradient echo sequence with Flip Angle: 10°; Repetition Time: 100 ms; Echo Time: 0.9s and voxel size: 1.8 × 1.8 × 5.0 mm, more details can be found at Table S2 (MERGEp); (**c**) Result of the automatic segmentation and labeling process of each tube on a cross section (each color corresponds to a label) of **1**; (**d**) Coding scheme of the different concentrations, the number of each concentration identifies the tube.

The methodology was based on measuring the relaxation rate (R expressed in s^−1^), which was obtained for each concentration by means of the computation of the corresponding relaxation time T of the studied phantoms (Figure 6). 

In the case of r_1_, it was obtained by calculating the T1 time from sequences with 2, 5, 10, 15, 25, and 45 flip angles, whereas r_2_ and r_2_* values were obtained after computing T2 and T2* relaxation times, which came from sequences with echo times indicated in Table S2. 

Thus, the longitudinal relaxivity (r_1_) for this compound based on pyridoxine at 3 T was determined to be 34.8 mM^−1^s^−1^, whereas the transversal relaxivities r_2_ and r_2_* values were 18.4 and 16.6 mM^−1^s^−1^, respectively (Figure S2). These experimental results obtained for **1** show relaxivity values which are somewhat higher than those previously reported for other of our compounds, namely, the [Gd(thy)_2_(H_2_O)_6_](ClO_4_)_3_·2H_2_O compound (thy = thymine), which displays r_1_, r_2_, and r_2_* values of 16.1, 13.5, and 14.5 mM^−1^s^−1^ at 3 T, respectively [30]. In any case, these two Gd^III^ compounds based on biomolecules exhibit relaxivity values larger than those of commercial MR imaging contrast agents, such as Gadovist, Prohance, Dotarem, Omniscan, and Magnevist [40,41,42,43,44,45]. These experimental features make **1** potentially useful for further MRI studies, the next step being in vitro investigations. 

## 3. Materials and Methods

### 3.1. Preparation of the Complex

#### Synthesis of **1**

A mixture of GdCl_3_·6H_2_O (92.9 mg, 0.25 mmol) and pyridoxine (84.6 mg, 0.50 mmol) was dissolved in EtOH (3 mL) and was stirred and heated at 60 °C for 1 h. The resulting solution was filtered and then poured in a test tube and layered with n-hexane. Colorless crystals of **1** were obtained in one week and were suitable for data collection of single-crystal X-ray diffraction studies. Yield: ca. 55%. Elemental analysis calculated (found) for C_16_H_34_N_2_O_12_Cl_3_Gd (**1**): C, 27.1 (26.8); H, 4.8 (4.8); N, 3.9 (3.7)%. SEM-EDX analysis gave a Gd:Cl molar ratio of 1:3 (Figure S3). ESI-MS (*m*/*z*): 567.75 (95%). UV-vis peaks were obtained at (nm): 220, 254 and 325. These values support the stability of complex **1** in solution (Figure S4 and Figure S5). Selected IR data (in KBr/cm^−1^): peaks were obtained at 3279 (s), 3172 (s), 3059 (m), 2877 (m), 2688 (m), 1617 (m), 1560 (m), 1441 (s), 1415 (m), 1352 (m), 1284 (m), 1222 (vs), 1084 (m), 1023 (vs), 984 (m), 958 (m), 885 (m), 757 (s), 694 (m), 574 (s), 519 (w), 486 (w), and 417 (m) (Figure S6).

### 3.2. X-ray Data Collection

Our X-ray diffraction data collection was performed on a selected crystal of **1** with a size of 0.14 × 0.10 × 0.05 mm^3^. This crystal was measured on a Bruker D8 Venture diffractometer with PHOTON II detector at 120 K. The radiation employed was that of Mo-K_α_ with λ = 0.71073 Å. In Table 1, the results of refinement along with the crystal parameters for **1** are tabulated. The crystal structure of **1** was solved through of direct methods and the SHELXTL program [46]. The graphical manipulations were performed by means of the DIAMOND program [47]. The H atoms of the pyridoxine ligands were located in computed positions and refined isotropically through the riding model. The H atoms were found on all the reported water molecules and were fixed through DFIX. 2311814 is the CCDC number for the crystal structure of **1**.

### 3.3. Technical Equipment, Devices and Physical Measurements

An elemental analyzer CE Instruments CHNS1100 was employed to carry out elemental analyses on samples of compound **1**. A Hitachi S-4800 field emission scanning electron microscope generated the results of scanning electron microscopy (SEM-EDX). A SCIEX TripleTOF 6600+ mass spectrometer was used to obtain the Electrospray Ionization Mass Spectrometry (ESI-MS) spectrum of **1**. All these technical measurements were carried out in the Central Service for the Support to Experimental Research (SCSIE) at the University of Valencia (UV). Next, Infrared (IR) and UV-vis spectra of **1** were recorded with a PerkinElmer Spectrum 65 FT-IR and a V-670 (Jasco Deutschland GmbH, Pfungstadt, Germany) spectrometers in the 400–4000 cm^−1^ and 200–800 nm regions, respectively. In addition, two magnetometers, namely, a Quantum Design MPMS-XL SQUID and a Physical Property Measurement System (PPMS), were used by us to perform magnetic susceptibility measurements, in both dc and ac, at the Institute of Molecular Science (ICMol-UV). During these magnetic measurements, we used eicosene to immobilize the investigated sample. Finally, the diamagnetic contribution of the involved atoms of compound **1** was corrected through Pascal’s constants method [48].

## 4. Conclusions

In summary, the preparation, crystallographic studies, and magnetic and relaxometric properties of a novel mononuclear Gd^III^ complex based on pyridoxine molecule and formula [Gd^III^(pyr)_2_(H_2_O)_4_]Cl_3_ · 2 H_2_O (**1**) [pyr = pyridoxine] were reported. 

This compound crystallizes in the triclinic space group *P*ī and its crystal packing exhibits a network of H-bonding interactions involving cationic units connected through non-coordinating water molecules and chloride anions. These intermolecular interactions were further investigated by means of its Hirshfeld surfaces. In addition, selected crystal structural data were used to be computed by means of the SHAPE program, whose results account for a triangular dodecahedron geometry (TDD) and a D_2d_ symmetry assigned to the Gd^III^ metal ion in compound **1**. 

The study of the magnetic properties of **1**, through both dc and ac magnetic susceptibility measurements, reveals a behavior typical of a quasi-isotropic metal ion displaying field-induced slow relaxation of magnetization and single-ion magnet (SIM) phenomenon. This magneto-structural study carried out on compound **1** is the first one performed on a lanthanide-based complex obtained with pyridoxine molecule, this fact indicating that the preparation and study of the magnetic properties of pyridoxine complexes with other more anisotropic lanthanide(III) ions, such as Tb^III^, Dy^III^, and Ho^III^, could generate an interesting family of SIMs based on this versatile biomolecule. This investigation is underway in our research group.

Finally, the relaxivity properties of **1** were investigated through a preliminary study carried out by means of magnetic resonance (MR) images of the tube phantoms containing different concentrations of complex **1** prepared in physiological serum. These images were collected on a 3T clinical MRI scanner. Our results indicate that complex **1** exhibits high relaxivity values in comparison with some currently used commercial contrast agents. Hence, **1** can be considered as a potential contrast agent for high-field MR imaging and a suitable candidate for further developments and MR imaging studies on this biomedical research field.

## Figures and Tables

**Table 1:** Summary of the crystal data and structure refinement parameters for compound **1**.

Compound	1
CCDC	2311814
Formula	C_16_H_34_N_2_O_12_Cl_3_Gd
Fw/g mol^−1^	710.05
Crystal system	Triclinic
Space group	*P*ī
*a*/Å	9.042(1)
*b*/Å	9.323(1)
*c*/Å	16.265(1)
α/°	79.92(1)
β/°	76.68(1)
γ/°	84.09(1)
*V*/Å^3^	1310.9(1)
*Z*	2
*D*_c_/g cm^−3^	1.799
μ(Mo-K_α_)/mm^−1^	2.894
*F*(000)	710
Goodness-of-fit on *F*^2^	1.068
*R*_1_ [*I* > 2σ(*I*)]/all data	0.0174/0.0199
w*R*_2_ [*I* > 2σ(*I*)]/all data	0.0382/0.0403

**Table 2:** Selected hydrogen-bonding interactions in **1** ^a^.

D-H···A	D-H/Å	H···A/Å	D···A/Å	(DHA)/°
O2-H2B···O5w	0.840	1.78(1)	2.607(1)	169.0(1)
O5-H5B···Cl1	0.840	2.27(1)	3.064(1)	159.0(1)
O1w-H1wA···Cl3f	0.939	2.25(1)	3.191(1)	176.7(1)
O1w-H1wB···Cl2a	0.940	2.21(1)	3.133(1)	166.3(1)
O2w-H2wA···Cl2	0.938	2.20(1)	3.103(1)	161.0(1)
O2w-H2wB···O3b	0.943	1.80(1)	2.730(1)	169.0(1)
O3w-H3wA···Cl2	0.935	2.40(1)	3.311(1)	165.4(1)
O3w-H3wB···Cl1e	0.940	2.12(1)	3.064(1)	178.7(1)
O4w-H4wA···Cl2a	0.940	2.24(1)	3.147(1)	162.4(1)
O4w-H4wB···O6e	0.940	1.80(1)	2.743(1)	178.6(1)
O5w-H5wB···Cl3c	0.944	2.21(1)	3.146(1)	173.5(1)
O6w-H6wA···Cl3	0.948	2.16(1)	3.107(1)	174.7(1)
O6w-H6wB···Cl1g	0.943	2.25(1)	3.113(1)	152.6(1)
N1-H1A···Cl3d	0.880	2.40(1)	3.218(1)	155.3(1)
N2-H2A···O6w	0.880	1.79(1)	2.640(1)	162.7(1)
O6-H6A···O4f	0.892	1.99(1)	2.749(1)	161.2(1)
O3-H3A···Cl3h	0.888	2.39(1)	3.139(1)	159.6(1)
O5w-H5wA···Cl1e	0.955	2.25(1)	3.165(1)	159.4(1)

^a^ Symmetry codes: (a) = x + 1, y, z; (b) = −x + 1, −y + 2, −z; (c) = −x + 1, −y + 2, −z + 1; (d) = x + 1, y, z − 1; (e) = x, y + 1, z; (f) = −x + 1, −y + 1, −z + 1; (g) = −x, −y + 1, −z + 1; (h) = x + 1, y + 1, z − 1.

**Table 3:** Selected values obtained through the SHAPE program for possible geometries with coordination number (CN) equal to 8 and from the bond lengths of compound **1** ^a^.

HPY	HBPY	CU	SAPR	TDD	JGBF	JETBPY	BTPR	JSD	TT
23.736	15.589	11.208	1.441	0.948	13.244	28.568	1.632	2.696	11.950

^a^ Heptagonal pyramid (HPY); Hexagonal bipyramid (HBPY); Cube (CU); Square antiprism (SAPR); Triangular dodecahedron (TDD); Johnson gyrobifastigum (JGBF); Johnson elongated triangular bipyramid (JETBPY); Biaugmented trigonal prism (BTPR); Snub diphenoid (JSD); Triakis tetrahedron (TT).

## Data Availability

The raw data that support the findings of this study are available from the corresponding author upon reasonable request.

## Supplementary Materials

The following supporting information can be downloaded at: https://www.mdpi.com/article/10.3390/ijms25042112/s1.
