# Supplementary material for: A Gadolinium(III) Complex Based on Pyridoxine Molecule with Single-Ion Magnet and Magnetic Resonance Imaging Properties
Source: Int J Mol Sci. 2024 Feb 9;25(4):2112. doi: 10.3390/ijms25042112 (PMC10889197; doi:10.3390/ijms25042112)
Supplement: Supplementary file 1 [file ijms-25-02112-s001.zip › ijms-2799913-supplementary.pdf]

Supplementary Information

A gadolinium(III) complex based on pyridoxine molecule with single-ion magnet and magnetic resonance imaging properties

Marta Orts-Arroyo,[a] Amadeo Ten-Esteve,[b] Sonia Ginés-Cárdenas,[b] Leonor Cerdá-Alberich,[b] Luis Martí-Bonmatí[b] and José Martínez-Lillo[a,*]

[a] *Instituto de Ciencia Molecular (ICMol), c/ Catedrático José Beltrán 2, 46980, Paterna, València, Spain. E-mail: f.jose.martinez@uv.es*

[b] *Radiology and Biomedical Imaging Research Group (GIBI2[30]), La Fe University and Polytechnic Hospital and La Fe Health Research Institute, Valencia, Spain. E-mail: luis_marti@iislafe.es*

Table of contents **page**

Table S1..2

Figure S1..3

Table S2..4

Figure S2..4

Figure S3..5

Figure S4..5

Figure S5..6

Figure S6..6

Table S1. Selected bond lengths (Å) for compound **1**.

Compound	**1**
Gd(1)-O(1)	2.255(1)
Gd(1)-O(2)	2.433(1)
Gd(1)-O(4)	2.308(1)
Gd(1)-O(5)	2.444(1)
Gd(1)-O(1w)	2.423(1)
Gd(1)-O(2w)	2.410(1)
Gd(1)-O(3w)	2.414(1)
Gd(1)-O(4w)	2.449(1)
O(1)-C(2)	1.303(2)
O(2)-C(7)	1.441(2)
O(3)-C(8)	1.432(2)
O(4)-C(10)	1.313(2)
O(5)-C(15)	1.436(2)
O(6)-C(16)	1.424(2)
C(1)-N(1)	1.343(2)
C(5)-N(1)	1.352(2)
C(9)-N(2)	1.343(2)
C(13)-N(2)	1.349(2)
C(1)-C(2)	1.415(2)
C(1)-C(6)	1.485(2)
C(2)-C(3)	1.415(2)
C(3)-C(4)	1.400(2)
C(3)-C(7)	1.502(2)
C(4)-C(5)	1.381(2)
C(4)-C(8)	1.506(2)
C(9)-C(10)	1.415(2)

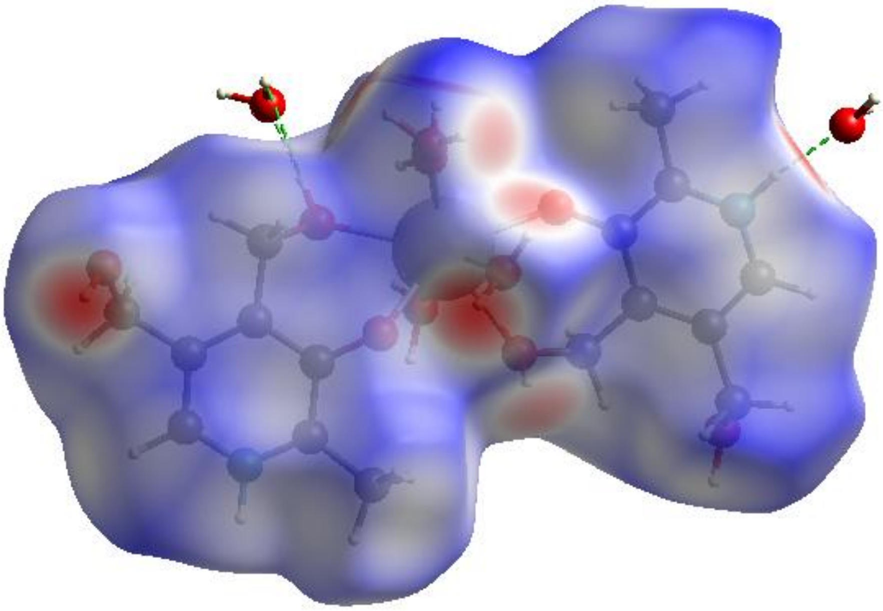

Figure S1. Hirshfeld surface mapped with d_{norm} function, showing the shorter intermolecular H···O contacts (dashed lines) between alcohol and N-H groups and non-coordinated water molecules for **1**.

Table S2. Parameters of the three main sequences to obtain the maps of T2, T2* and T1, respectively. Subindex p of METSEp, MEGREp and MAPp refers to the sequences used in the Philips Achieva (3T) equipment for compound **1**.

	METSEp	MEGREp	MAPp
Scanner	Philips Achieva	Philips Achieva	Philips Achieva
FA [°]	90	10	2,5,10,15,25,45
TR [ms]	5000	100	14
TE_1 [ms]	10	0.9	4.6
nTE [a.u.]	32	32	1
ΔTE [ms]	10	0.7	NA
Voxel Size [mm]	1.8	1.8	1.8
Slice Thickness [mm]	5	5	5
FOV [mm]	230x230	230x230	288x288
N Slices [a.u.]	1	15	15
Duration [s]	315	75	5 x 64

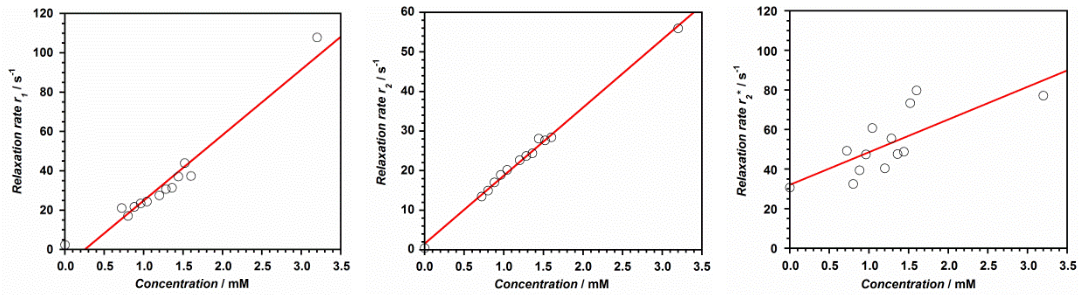

(a) (b) (c)

Figure S2. Relaxation rate vs. contrast concentration plot obtained for the relaxivities r_1 (a), r_2 (b) and r_2* (c) of compound **1**. The red line represents the best linear fit of the experimental data.

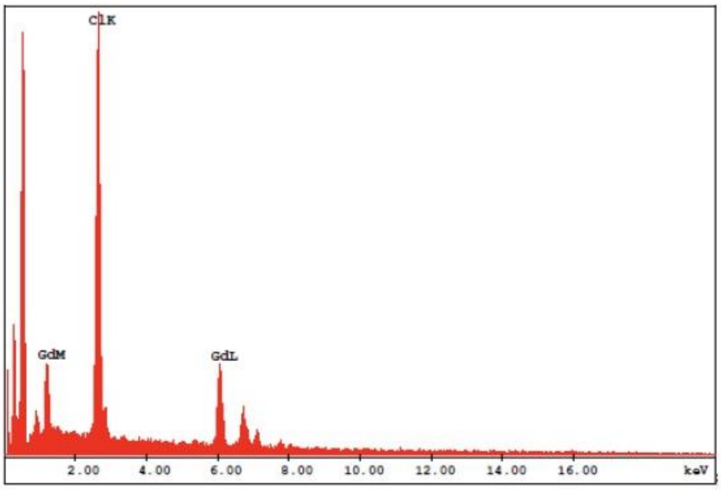

Figure S3. SEM-EDAX spectrum for compound **1**.

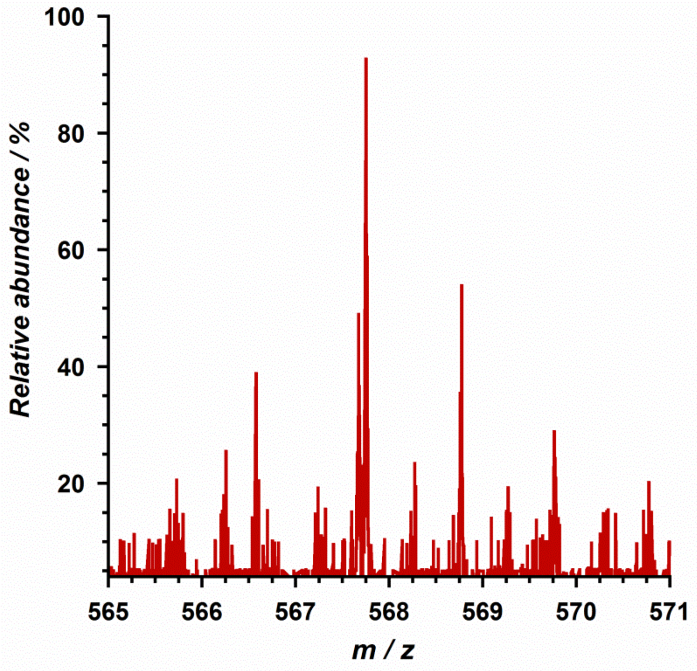

Figure S4. Electrospray ionization mass spectrum (ESI-MS) for compound **1** showing the isotopic distribution for the $[Gd(pyr)_2(H_2O)_4]^{3+}$ cation.

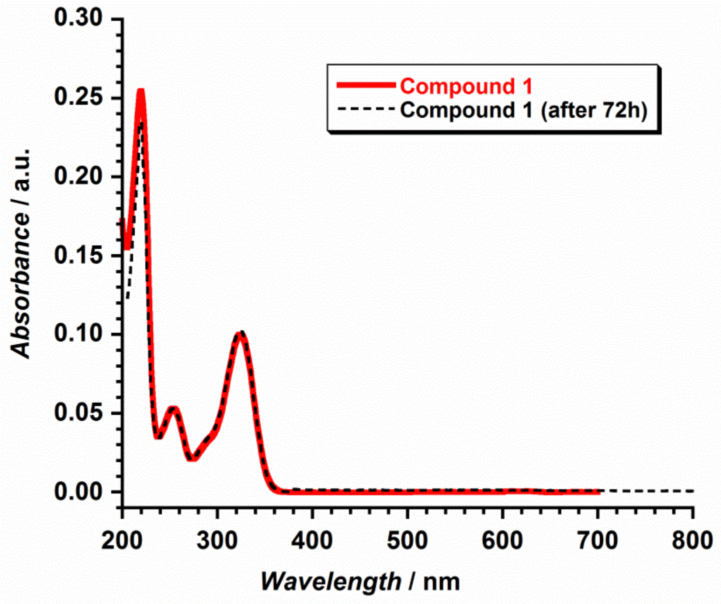

Figure S5. Absorption spectra (Abs. versus λ) covering the range of 200–800 nm for compound **1** (solid red line) and for compound **1** after 72h (dashed black line).

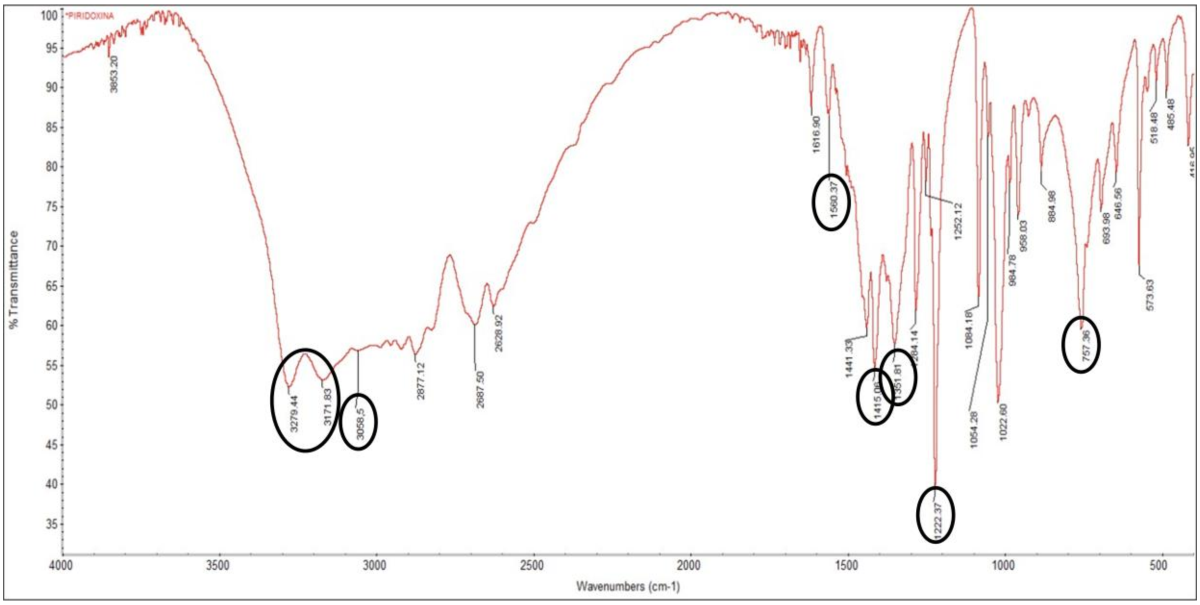

Figure S6. FT-IR spectrum for compound **1**.
